# The first Saudi Arabian national inventory study revealed the upcoming challenges of highly diverse non-tuberculous mycobacterial diseases

**DOI:** 10.1371/journal.pntd.0006515

**Published:** 2018-05-25

**Authors:** Bright Varghese, Mushira Enani, Mohammed Shoukri, Sameera AlJohani, Hawra Al Ghafli, Sahar AlThawadi, Sahal Al Hajoj

**Affiliations:** 1 Department of Infection and Immunity, King Faisal Specialist Hospital and Research Centre, Riyadh, Saudi Arabia; 2 Medical Specialities Department, King Fahad Medical City, Riyadh, Saudi Arabia; 3 National Biotechnology Centre, King Faisal Specialist Hospital and Research Centre, Riyadh, Saudi Arabia; 4 Department of Pathology and Laboratory Medicine, King Abdulaziz Medical City, Riyadh, Saudi Arabia; 5 Department of Pathology and Laboratory Medicine, King Faisal Specialist Hospital and Research Centre, Riyadh, Saudi Arabia; Hospital Infantil de Mexico Federico Gomez, UNITED STATES

## Abstract

**Background:**

Incidences of nontuberculous mycobacteria (NTM) causing pulmonary and extrapulmonary diseases are reportedly increasing globally and the current epidemiologic situation in Saudi Arabia remains unclear. To study such trend, we carried out a nationwide systematic epidemiological study focusing on NTM diseases for the first time in the country.

**Methods/Principle findings:**

A nationwide collection of NTM isolates with clinical and demographical data was conducted for a period of 24 months. Primary species identification was carried out by line probe assays followed by sequencing of *16S rRNA*, *16S-23S ITS region*, *rpoB* and *hsp65* genes. The laboratory findings were comprehensively analysed against demographical and clinical data. A total of 527 isolates were enrolled with a higher proportion of Saudi citizens (76.5%), elderly (>60 years) patients (34.2%), and male gender (65.3%) respectively. Overall, 75.1% isolates were pulmonary origin with a proven clinical significance of 44.7%. In total, 34 NTM species including 17 rare species were identified, in addition to 8 ‘undefined’ isolates. *M*.*simiae* (22.6%), *M*.*fortuitum* (18.1%) and *M*.*abscessus* (17.8%) were predominant species. Interestingly, 27 new cases of clinically relevant *M*.*riyadhense* were also noticed (Primary data on emergence of rare NTM species and *M*.*riyadhense* has been recently reported). Results showed, rare clinical events such as mycobacteremia, cecum abscess, peritonitis and ascites caused by *M*.*wolinskyi*, *M*.*holsaticum*, *M*.*duvalii* and *M*.*monacence* respectively. Diabetes mellitus (P value-0.04) and previous history of tuberculosis (P value- 0.001) were identified as independent risk factors associated with NTM diseases.

**Conclusions/Significance:**

NTM disease spectrum and pathogen diversity is an emerging challenge to any nation, including Saudi Arabia. Therefore, more priorities will be given to NTM’s with an immediate initiative to develop diagnostic infrastructures and disease management plans.

## Author summary

Non-tuberculous mycobacteria (NTM) are opportunistic pathogens and saprophytes found largely in wet soil, marshland, streams, rivers and estuaries. Pulmonary and extrapulmonary Infections caused by NTM’s increased globally in immune-suppressed and immune-competent individuals without an exemption to Saudi Arabia. However, recent developments in diagnostic platforms improved the identification of several new and rare species of NTM’s. Interestingly, recent studies linked the direct impact of species spectrum on clinical outcome and therapies. To date, in Saudi Arabia, no systematic nationwide surveillance study was conducted on the challenges of NTM causing diseases. Therefore, for the first time, a nationwide inventory study on the spectrum of clinically relevant pulmonary and extra pulmonary diseases with an attempt to explore epidemiological data has been carried out. The study results showed a huge species spectrum of NTM’s causing highly diverse clinical manifestations. Clinical relevance pulmonary infection was relatively high and several very rare extrapulmonary infections also were observed. The study reported the emergence of a rare pathogen, *Mycobacterium riyadhense* and predominance of *Mycobacterium simiae* as the most common species. As the first large scale study from the GCC region, the findings cements the growing potential of various NTM species to cause significant clinical implications in the country and warrants an immediate change on disease management priorities.

## Introduction

Globally, pulmonary and extrapulmonary human infections caused by various non-tuberculous mycobacteria (NTM) were reportedly increased [1–5]. Although, immunosuppressive illnesses and therapies are regarded as major predisposing factors, there was recent increase of NTM incidences among immunocompetent patients. Despite this rise, NTM diseases are not yet considered as serious public health threat and these diseases remain largely neglected in most parts of the world. Unlike tuberculosis, NTM causing diseases are not notifiable conditions in most of the countries, except in regions like Australia [6]. Scarcity of evidences to support human-to-human transmission and the facultative nature of NTM entities have contributed remarkably towards such negligence. To date, almost 150 species of NTM’s are officially recognized as per the List of Prokaryotic Names with Standing in Nomenclature (http://www.bacterio.net/mycobacterium.html). The pathogenic potential of most of the species is still not fully defined and hardly few dozen are only familiar to clinicians and microbiologist [7]. Species spectrum of NTM’s are mostly associated with geographical distribution, thus the diversity varies from region to region [8].

Approximately, 75–90% of NTM’s are isolated from pulmonary samples, however majority remains as colonizers [9]. Extrapulmonary involvement had been commonly observed in lymphnode, skin, soft tissues, bone and less frequently in central nervous, urogenital and gastro intestinal systems [10]. Regardless of the broad disease spectrum, data on definitive NTM incidences are scarce in most part of the world, particularly from developing countries.

To date, no nationwide systematic epidemiological study on clinical relevance of NTM’s was conducted in Saudi Arabia. However, recently Varghese et al. showed, considerable threats from several NTM species causing pulmonary and extrapulmonary diseases [11, 12]. Therefore, for the first time a prospective study has been carried out to analyse the species spectrum, clinical relevance, underlying risk factors, possible mortality and geographical adaptation of NTM’s in the country.

## Materials and methods

### Ethics statement

This study has been reviewed and approved by the Office of Research Affairs of King Faisal Specialist Hospital and Research Centre, Riyadh.

### Study population and settings

During April-2014 to March-2016, 527 non repetitive NTM isolates were collected from different provincial laboratories. The sample volume has been calculated following a previously published national data on NTM isolation [13]. Clinical and demographical data including data on previous NTM isolations have been archived from medical and laboratory records. The study had two major case groups; pulmonary (396 isolates) and extrapulmonary (131 isolates). Clinical relevance of pulmonary group was defined according to the American Thoracic Society (ATS) guidelines [9]. Briefly, clinical relevance was confirmed if cases meet any of the following criteria, culture positivity detected in a minimum of two separate sputum or tracheal aspirates; one lung tissue with histopathological relevance; or bronchio-alveolar lavage. All the extrapulmonary cases were considered as clinically relevant. Mortality data has been collected after referring to the laboratory records at the end of sample collection period (month 22–24) from each study sites.

### Identification of isolates

Isolates were primarily identified by using line probe assays (Mycobacterium CM and AS -Hain Life Science, Germany). Isolates which identified only up to *Mycobacterium species* level were subjected to sequencing of four highly conserved genes, *16S rRNA*, *rpoB*, *16S-23S ITS region* and *hsp65* using the BigDye Terminator cycle sequencing chemistry (Applied Biosystems, USA). Primary sequencing was targeted on *16S rRNA* (645-655bp hyper variable region) and a 342bp region of *rpoB* genes, followed by *hsp65* (439bp) and *16S-23S ITS region* (480bp) genes [14–17].

### Data analysis

#### Case definitions

Rare species: Isolates which could not be identified up to species level by line probe assays alone and identified only by gene sequencing were defined as “rare species”.

Risk factors: Risk factor in the study was defined with a broad spectrum, that any direct attribute on demographical features (age, gender, geographical zone of origin) or any underlying disorders which chronically impair immune host response with a probabilistic effect on the likelihood of NTM disease were considered as risk factor in current analysis.

Elderly population: Patients aged above 60 years were grouped as “elderly” in the cohort regardless of nationality.

The sequence base calling was carried out in Sequence Analysis software (Applied Biosystems, USA) and assembled in Seqman-Pro (DNA STAR, USA). Assembled sequences were subjected to BLAST analysis in NCBI GenBank and EzTaxon (http://www.ezbiocloud.net/identify) databases. A stringent similarity index ≥99–100% was followed with Type strain in the database. Isolates which could not be identified even after performing sequencing were assigned as *Mycobacterium species*. All the statistical data analysis was carried out by using SPSS-V20.0 software package (IBM, USA). We used the linear regression analysis for analysing the risk factors of NTM disease in the country stratified by the origin of patients. A P value <0.05 was considered as statistically significant.

## Results

### Demographical and clinical findings

A total of 527 isolates from different geographical region were collected with a demographical predominance of male gender (65.3%), age group elderly (34.2%) and Saudi nationals (76.5%). Geographical case distribution showed more incidences in West (33.8%) followed by Central (30%) and East (22.2%) (Table 1).

**Table 1:** Demographical and clinical summary of study population.

Parameters	No/%
**Gender**	
Male	344(65.3)
Female	183(34.7)
**Age Group**	
1–15	35(6.6)
16–29	80(15.2)
30–45	108(20.5)
46–59	124(23.5)
≥60	180(34.2)
**Nationality**	
Saudi	403(76.5)
Non-Saudi	124(23.5)
**-South East Asian**	25(20.2)
**-Indian subcontinent**	28(22.6)
**-South American**	2(1.6)
**-European**	4(3.2)
**-African**	45(36.3)
**-Middle Eastern**	20(16.1)
**Provincial Origin of patients**	
South	64(12.1)
East	117(22.2)
**West**	178(33.8)
Central	158(30)
North	10(1.9)
**Site of Infection**	
Pulmonary	**396(75.1)**
-Clinically relevant	177(44.7)
**-Colonizer**	219(55.3)
Extrapulmonary	**131(24.9)**
**AFB smear**	
Positive	391(74.2)
Negative	136(25.8)
**HIV**	
Positive	14(2.6)
Negative	412(78.2)
**Treatment Received**	178(33.8)
**Died**	12(2.3)
Pulmonary ^a^	7 (58.3)
Extra-pulmonary ^b^	5(41.7)

a Caused by *M.abscessus*-4, *M.simiae*-3

b Caused by *M simiae* only

**Fig 1:**
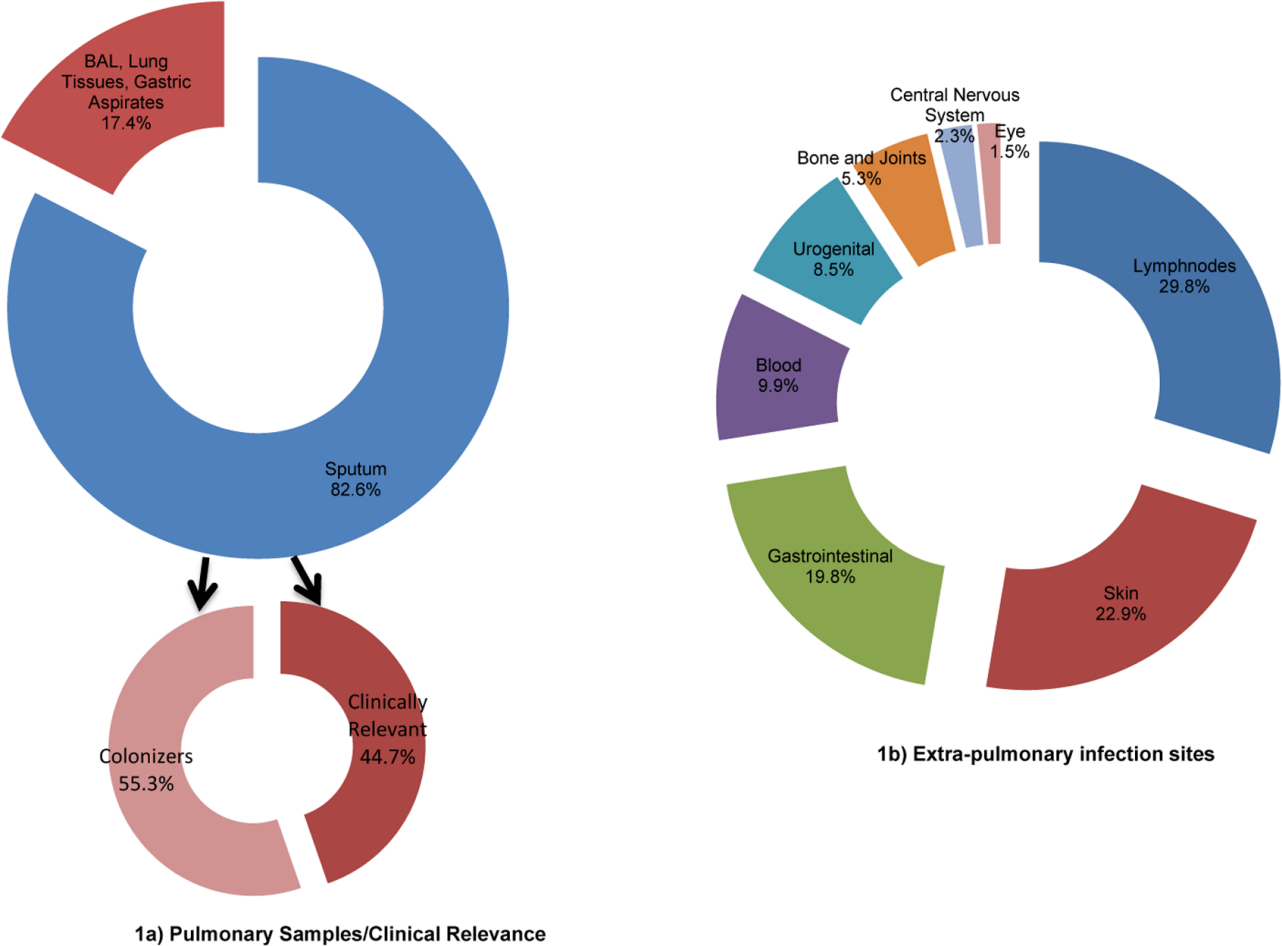
Proportion of pulmonary and extrapulmonary site of infections in the study. This figure shows the source of NTM isolation in the study from different pulmonary and extrapulmonary clinical samples.

Pulmonary isolates were dominant (75.1%) and 82.6% of pulmonary NTM isolation was from sputum samples. Following ATS guidelines, a clinical relevance of 44.7% and 55.3% colonization were observed. Lymphnode (29.8%) and skin (22.9%) were the most affected extrapulmonary sites. Interestingly, 13(9.9%) cases of mycobacteremia and 17.6% of various other infections also reported (Fig 1).

Only, 426(80.8%) cases were tested for HIV and reported with 14(3.3%) positive cases. Overall, 178(33.8%) cases received treatment and most among them had extrapulmonary diseases (129/131; 98.5%) (Table 1).

### Species diversity of NTM’s

**Fig 2:**
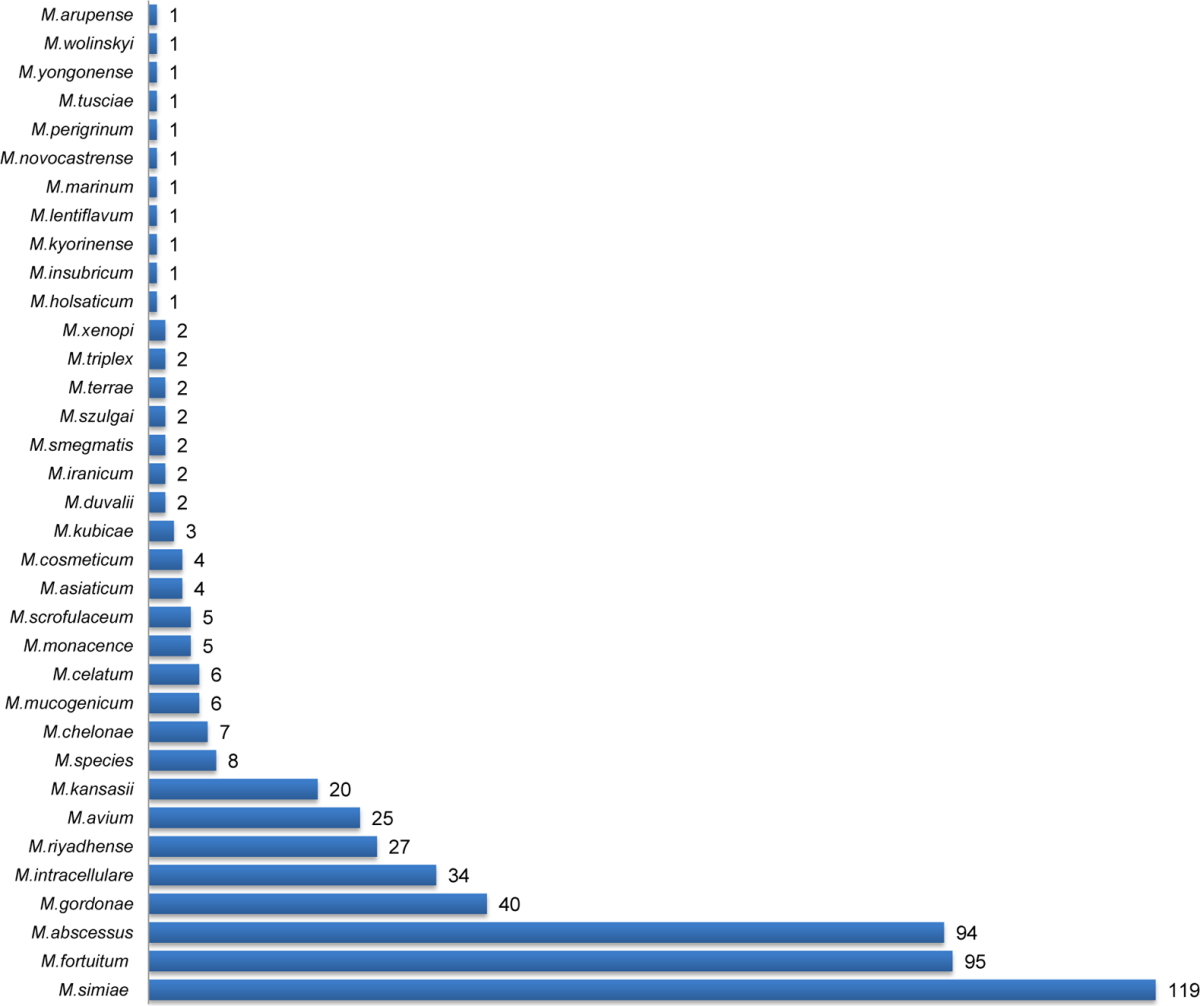
Species spectrum of 527 clinically isolated NTM’s in Saudi Arabia. The figure shows the overall species diversity in the study detected by line probe assays and gene sequencing.

A total of 34 different species including 17 rarely reported species were identified. The slow growing species (56.7%) were predominant with an increased isolation of *M*.*simiae* (22.6%). *M*.*fortuitum* (18.1%), *M*.*abscessus* (17.8%) and *M*.*gordonae* (7.6%) were the other major species isolated. Overall, 29(5.5%) isolates were identified as ‘rare’ species, which mainly included *M*.*monacence* and *M*.*cosmeticum*. *M*.*riyadhense* was isolated from 27 patients and 8(1.5%) isolates were identified only up to *Mycobacterium species* (Fig 2). Published primary findings of the study revealed existence of 12 *M*.*riyadhense* and 27 ‘rare’ NTM cases [11, 18].

### NTM species and site of infections

*M*.*avium*, *M*.*riyadhense* and *M*.*gordonae* were frequently isolated from pulmonary samples. However, most of the identified species (20/34 species) were involved in different extrapulmonary anatomical sites. *M*.*fortuitum* (22.1%), *M*.*abscesuss* (21.4%) and *M*.*simiae* (19.1%) were the predominant in extrapulmonary infections. Interestingly, 75(57.2%) isolates were rapid growers, including 6 rare species (*M*.*holsaticum*, *M*. *duvalii*, *M*.*monacense*, *M*.*wolinskyi*, *M*.*peregrinum*, and *M*. *cosmeticum*). The analysis showed rare clinical events such as mycobacteremia, cecum abscess, peritonitis and ascites caused by *M*.*wolinskyi*, *M*.*holsaticum*, *M*.*duvalii* and *M*.*monacence* respectively (Table 2).

**Table 2:** Proportion NTM species causing extrapulmonary disease against anatomic site of infections.

Sites	Total cases	*M*.*abscessus*	*M*.*fortuitum*	*M*.*simiae*	*M*.*avium*	*M*.*intracellulare*	*M*.*kansasii*	*Others*
**Lymphnode**	39	8(20.6)	7(17.9)	5(12.8)	1(2.6)	7(17.9)	1(2.6)	10(25.6)^a^
**Skin/abscess**	30	4(13.3)	8(26.7)	8(26.7)		1(3.3)	2(6.6)	7(23.4) ^b^
**Blood**	13	2(15.4)	2(15.4)	4(30.8) ^k^	1(7.6)			4(30.8)^c^
**Ascitic fluid**	6		2(33.3)					4(66.7)^d^
**Peritoneal fluid**	8	2(25)	3(37.5)	1(12.5) ^l^			1(12.5)	1(12.5)^e^
**Liver biopsy**	3	1(33.3)	1(33.3)		1(33.3)			
**Bone/ Joints**	7	4(57.1)		1(14.3)				2(28.6) ^f^
**Intestine**	5	1(20)	1(20)	1(20)	1(20)			1(20)^g^
**Cecum**	2			1(50)				1(50)^h^
**Colon**	4	1(25)	1(25)	1(25)				1(25)^i^
**Pleural fluid**	6	1(16.7)	3(50)	1(16.7)			1(16.7)	
**Eye**	2	1(50)	1(50)					
**CSF**	3	1(33.3)		1(33.3)^m^				1(33.3)^j^
**Urine**	3	2(66.7)		1(33.3)				
**Total**	131	28(21.4)	29(22.1)	25(19.1)	4(3.1)	8(6.1)	5(3.8)	32(24.4)

^a^ M.scrofulsceum(4), M.chelonae(2), M.riyadhense(1), M.cosmeticum(1), M.species(2).

^b^ M.chelonae(1), M.marinum(1), M.peregrinum(1), M.species(2), M.mucogenicum(1), M.smegmatis(1)

^c^ M.mucogenicum(2), M.wolinskyi(1), M.species(1)

^d^ M.monacence(3), M.gordonae(1)

^e^ M.duvalii(1)

^f^ M.riyadhense(2)

^g^ M.gordonae

^h^ M.holsaticum(1)

^i^ M.chelonae

^j^ M.xenopi

k 3 patients died

l patient died

m patient died

### Geographical distribution of NTM species

**Fig 3:**
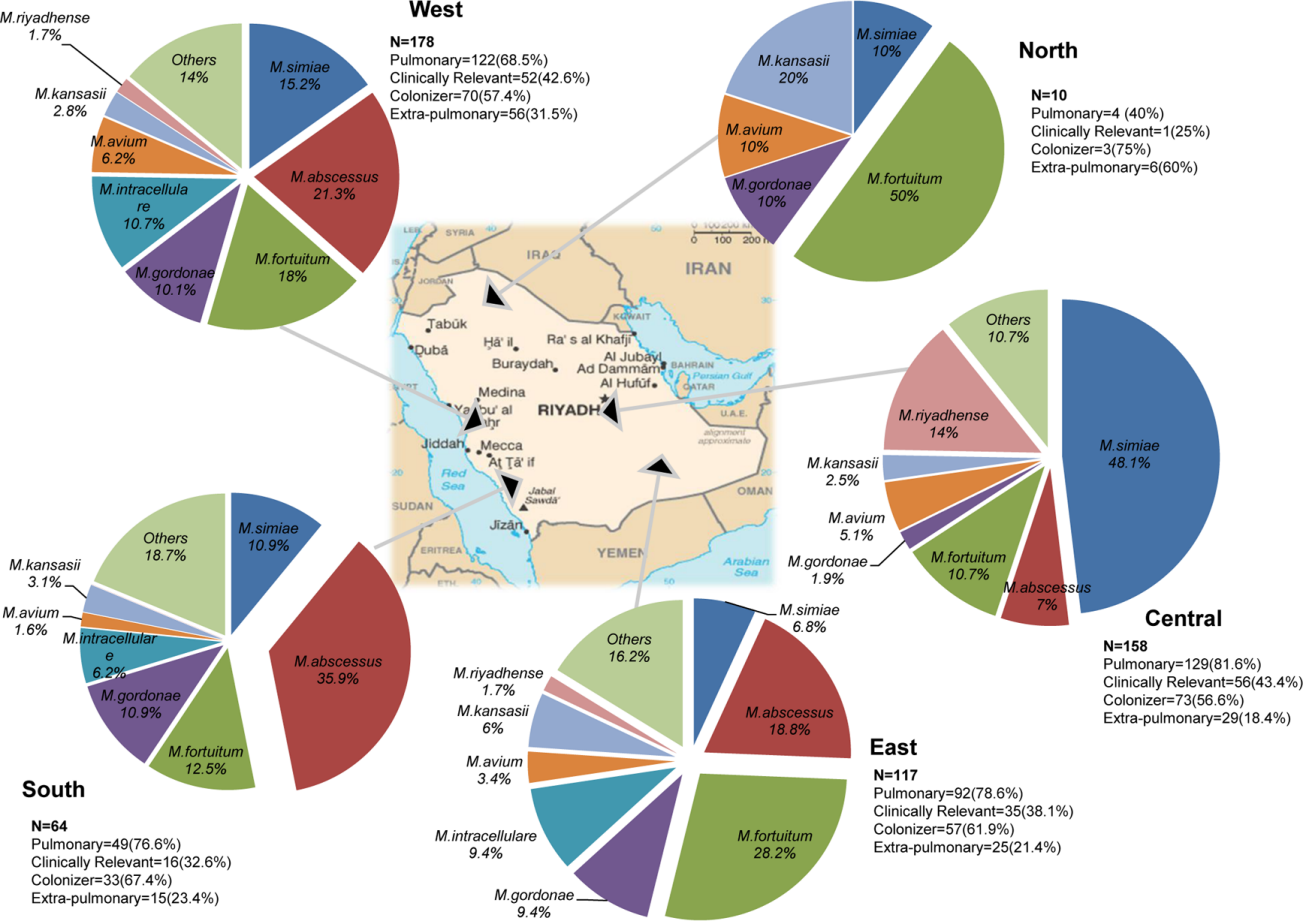
Geographical distribution and clinical relevance of major NTM species in Saudi Arabian provinces. This figure explains the distribution of the major NTM species in different geographical regions of Saudi Arabia. The used map of Saudi Arabia has been adapted from the open map resource https://commons.wikimedia.org/wiki/Atlas_of_Saudi_Arabia. ,.. Pie charts created and embedded by using MS Office Excel and Adobe Photoshop.

We analysed geographical distribution of 8 common species (*M*.*simiae*, *M*.*kansasii M*.*intracellulare*, *M*.*riyadhense*, *M*.*gordonae*, *M*.*abscessus*, *M*.*fortuitum*, and *M*.*avium)*. *M*.*abscessus* and *M*.*fortuitum* were commonly isolated from the West, South, and East regions. In Central province domination of *M*.*simiae* (48%) was evident with consistent isolation of *M*.*riyadhense*. Pulmonary cases were high in Central region (81.6%) while extrapulmonary in North (60%) and West (31.5%). Clinical relevance of pulmonary cases was observed high (43.4%) in Central province (Fig 3).

The Proportion of pulmonary and extrapulmonary infection in each region is also depicted. Clinical relevance and colonization of pulmonary NTM infections after consulting the American Thoracic Society guidelines also presented.

### Predisposing risk factors in NTM infected patients

Overall 29 different types of underlying conditions among 376 (71.3%) of cases were noticed including 39.4% cases with multiple immunosuppressive conditions. Majority of the confounding factors were recorded among Saudi population (80.5%). Diabetes mellitus (21.8%) was the most common among Saudi nationals. Smoking and hypertension were the other two factors found among both Saudi and non-Saudi nationals. There were five infectious diseases other than tuberculosis and HIV observed as underlying conditions (Table 3).

**Table 3:** Demographical and clinical risk factors associated with NTM infections in Saudi Arabian local and immigrant population.

	N(%)	Saudi	Non Saudi	OR(95% CI)	P value
**Gender**					
**Male**	344(65.3)	269(78.2)	75(21.8)	REF	
**Female**	183(34.7)	134(73.2)	49(26.8)	1.31(0.86–1.98)	0.20
**Age Group**					
**1–15**	35(6.6)	30(85.7)	5(14.3)	REF	
**16–29**	80(15.2)	51(63.7)	29(36.3)	3.41(1.19–9.75)	**0.02**
**30–45**	108(20.5)	52(48.1)	56(51.9)	6.46(2.33–17.91)	**0.003**
**46–59**	124(23.5)	95(76.6)	29(23.4)	1.83(0.65–5.15)	0.25
**≥60**	180(34.2)	175(97.2)	5(2.8)	0.17(0.04–0.63)	**0.007**
**Provincial Origin**					
**South**	64(12.2)	49(76.6)	15(23.4)	REF	
**East**	117(22.2)	88(75.2)	29(24.8)	1.07(0.53–2.19)	0.84
**West**	178(33.8)	132(74.1)	46(25.9)	1.13(0.58–2.22)	0.70
**Central**	158(29.9)	128(81)	30(19)	0.76(0.37–1.54)	0.45
**North**	10(1.9)	6(60)	4(40)	2.17(0.54–8.75)	0.27
**Site of Infection**					
**Pulmonary**	396(75.1)	304(76.8)	92(23.2)	REF	
**Extrapulmonary**	131(24.9)	99(75.6)	32(24.4)	1.06(0.67–1.69)	0.78
**Underlying conditions.**					
**COPD^a^**	20(3.8)	18(90)	2(10)	REF	
**Smoking**	54(10.2)	38(70.4)	16(29.6)	3.79(0.78–18.27)	0.09
**Diabetes mellitus**	115(21.8)	114(99.1)	1(0.9)	0.08(0.00–0.92)	**0.04**
**Hypertension**	48(9.1)	39(81.2)	9(18.8)	2.07(0.41–10.61)	0.38
**Carcinoma ^b^**	32(6.1)	31(96.9)	1(3.1)	0.29(0.02–3.43)	0.33
**Asthma**	39(7.4)	28(71.8)	11(28.9)	3.53(0.7–17.84)	0.12
**Rheumatoid Arthritis**	29(5.5)	26(89.6)	3(10.3)	1.04(0.16–6.87)	0.97
**ESRD ^c^**	26(4.9)	26(100)	-	0.14(0.00–3.08)	0.21
**IHD ^d^**	25(4.7)	24(96)	1(4)	0.37(0.03–4.46)	0.43
**Bronchiectasis**	19(3.6)	14(73.7)	5(26.3)	3.21(0.54–19.10)	0.19
**Previous TB disease**	83(15.7)	36(43.4)	47(56.6)	11.75(2.56–53.34)	**0.001**
**Lymphomas ^e^**	16(3.0)	16(100)	-	0.22(0.01–5.01)	0.34
**Leukemia**	14(2.6)	13(92.8)	1(7.2)	0.25(0.01–5.74)	0.39
**HIV**	14(2.6)	9(64.3)	5(35.7)	5.0(0.81–31.0)	0.08
**Others**	54(10.2)	41(75.9)	13(24.1)	-	-

Others (n): Ulcerative colitis(4), hypothyroidism(9), interstitial lung disease(9),multiple myeloma(2), empyema(2), Crohn’s disease(2), organ transplants(2), systemic lupus erythematous (2), severe combined immunodeficiency (2), Vitamin D deficiency (8), chronic liver disease (5), neutropenia(3), CD3 deficiency(2), bare lymphocyte syndrome(1), chronic granulomatous disease(1).

^a^ Chronic obstructive pulmonary disease

^b^ Intestine, liver, lungs, prostrate, breast, endometrium, pharynx, kidney.

^c^ End stage renal disease

^d^ Ischemic heart disease

^e^ Hodgkin’s and non-Hodgkin’s lymphomas

Among the demographical factors, the findings showed a clear statistical association of patients in age groups 16–29 (p-value, 0.02), 30–45 (p-value, 0.003) and >60 years (p-value, 0.007). Diabetes mellitus (p-value, 0.04) and previous history of tuberculosis disease (p-value, 0.001) were also statistically significant (Table 3).

### Mortality of NTM infected cases

Although detailed clinical follow ups were limited under the scope of the study, we analyzed the NTM mortality using laboratory records. Overall, 12(2.3%) patients died in the cohort due to pulmonary (58.3%) or extrapulmonary (41.7%) diseases (Table 1). Analysis of causative organism showed, presence of *M*.*sim*iae (66.7%) in most cases associated with extra pulmonary diseases including mycobacteremia. *M*.*abscessus* was found in other 4 (33.3%) died patients with pulmonary disease (Table 1, Table 2).

## Discussion

The study tried to explore the diversity and geographical distribution of various NTM species in Saudi Arabia with possible risk factors and related mortality. Demographical data revealed a higher proportion of NTM infection among Saudi nationals and particularly male, which is consistent with similar findings from recent but rather limited studies [11, 12]. Dominance of male gender is in concordance with a small study from Korea, while countries like USA showed female gender predominance in larger studies [1, 19]. The real reason behind such increased proportion among male gender is totally unclear. However, majority (80.5%) of Saudi patients have at least one underlying risk conditions including several cases with multiple comorbidities. The severe immunocompromised conditions such as lymphomas, carcinomas and leukaemia were mostly found among Saudi (>90%) patient cohort. Moreover, increased frequency of genetic predisposing conditions among the Saudi nationals mainly due to higher rate of consanguinity, increasing rate of different immunosuppressive therapies and various malignancies could be additional risks factors to increase the susceptibility to NTM infections [20–22]. Current data showed, individuals aged above 60 years were under particular risk of NTM infections, similar to studies reported with a higher isolation rate of NTM’s among elderly from Saudi Arabia and rest of the world [3, 12, 23]. Positive HIV cases were very much limited (2.6%) and comparably lower to other countries. It is worth mentioning that, the HIV prevalence among Saudi population (<4 cases/100000 populations) is very low and hence the same is reflected in our cohort [24].

Species spectrum of NTM was large, including several rare species. Emergence of *M*.*simiae* as the most common species was an unexpected finding, because none of the previous small studies reported the increasing prevalence of this species [12, 25]. The higher isolation (56.7%) of slow growing species is an opposite finding to a recent study which predominantly reported rapid growers (61.1%) in the country [12]. Interestingly, 16 rare species were observed including ‘very rare species (<10 cases in literature)’ such as *M*.*holsaticum*, *M*.*insubricum*, *M*.*tusciae*, *M*.*yongonense* and *M.kubicae [7]*. Although, rare in global distribution, *M*.*riyadhense* was considered as a common pathogen because the species recently established well in Saudi Arabia [18]. Furthermore, the “undefined” species may possibly identified into novel NTM species after detailed consultation of phenotypic and genotypic profiling, which in turn may maximize the current NTM species spectrum in the country.

Clinical relevance of pulmonary infections was high (44.7%) compared to previous studies [25, 26]. *M*.*simiae*, *M*.*fortuitum* and *M*.*abscessus* were commonly isolates from pulmonary samples, while clinical relevance of *M*.*simiae* (35.1%) and *M*.*abscessus* (27.3%) were low. *M*.*riyadhense* showed a higher clinical relevance of 83.3%. On the other hand, *M*.*avium* also showed a higher (66.7%) clinical relevance, which resembles similar findings from other part of the world [3, 23, 26].

Extrapulmonary infections sites showed variations in the proportion observed from previous international studies [4, 23]. Lymphnode was mostly affected, and such higher lymphnode infections rate is an opposite picture of other global regions [23]. In contrast, the NTM associated lymphadenopathy was found mainly among female (69.2%) and children (46.2%). *M*.*abscessus*, *M*.*scrofulaceum*, *M*.*intracellulare and M*.*fortutium* were the common causative agents for lymphadenopathy. Reported 10% NTM mycobacteremia with the presence of rare species such as *M*.*wolinskyi* and an ‘undefined species’ is a rising challenge to the health authorities [27]. Cecum infection caused by *M*.*holsaticum* and peritonitis caused by *M*.*duvalii*, were rare events which could be reported for the first time in literature.

Geographical distribution of different species showed varying prevalence. The major collection was carried in Western province, where *M*.*abscessus* and *M*.*fortuitum* were predominant, although similar trend was observed in Southern and Eastern provinces. All of these provinces are major regions for agriculture in the country, with several natural water sources. However, the central province has relatively different species diversity. *M*.*simiae* was dominant in the province followed by *M*.*riyadhense* and *M*.*fortuitum*. Central region is a landlocked province with lesser agriculture lands and limited water sources (mostly depending on desalination plants in other regions). We assume *M*.*riyadhense* is emerging as a new pathogen highly confined to the Central province of the country. *M*.*simiae* domination may probably relate to an outbreak as it was not evident in previous studies [12, 25]. Although, the sample size was small, Northern Province showed predominance of *M*.*fortu*itum followed by *M*.*kansasii*. The relatively low isolation may be linked to the overall low population density in the region.

Although, the reported size is small, this is the first report regarding the NTM associated mortality in Saudi Arabia. The reported mortality rate (2.3%) is comparatively lower than the reports from USA and Canada [28, 29]. However, the emergence of *M*.*simiae* as the major causative agent for disease leading to mortality needs further detailed investigations. The seemingly consistent impact of *M*.*abscesuss* in pulmonary NTM cases, often leading to death was previously established, and similar finding was currently observed [30]. However, due to the lack of detailed clinical follow up on study cases, the associated reasons of death and treatment details could not be well explained.

This study has few limitations to explain. Although, a very good representation of overall population had been taken, a pure population based epidemiological analysis was limited due to the laboratory targeted nature of the study and which restricted in-depth clinical data collection. Follow-ups of patients for a long period of time to assess the treatment outcome or more number of isolation to prove clinical significance of certain cases were limited. Finally, underlying co-morbid conditions of immigrant patients was highly scarce in records.

### Conclusions

In conclusion, Saudi Arabia currently faces several challenges from NTM diseases and they are generally neglected. The emergence of new pathogens such as *M*.*riyadhense* or difficult to treat species like *M*.*simiae*, should be addressed immediately. Presence of rare species of NTM’s which cause both pulmonary and extrapulmonary diseases to the Saudi population may show the potential adaptability of them in the country. Higher prevalence of Saudi population with NTM diseases must be investigated thoroughly to rule out the possibilities of NTM disease vulnerability. Finally, detailed management plan for mycobacterial disease control must be developed with urgent priorities.

## Supporting information

S1 STROBE Checklist (DOC)
